# A multivariate decomposition analysis of modern contraceptive utilization among married women in the emerging region of Ethiopia (2000–2019)

**DOI:** 10.1038/s41598-023-48176-4

**Published:** 2023-11-28

**Authors:** Natnael Kebede, Bereket Kefale, Muluken Yigezu, Kokeb Ayele, Elsabeth Addisu, Yawkal Tsega, Mulugeta Desalegn Kasaye, Yitbarek Wasihun

**Affiliations:** 1 Department of Health Promotion, School of Public Health, College of Medicine and Health Sciences, Wollo University, Dessie City, Ethiopia; 2 Department of Reproductive and Family Health, School of Public Health, College of Medicine and Health Sciences, Wollo University, Dessie City, Ethiopia; 3 Department of Public Health College of Medicine & Health Science, Dire Dawa University, Dire Dawa City, Ethiopia; 4 Department of Health System and Management, School of Public Health, College of Medicine and Health Sciences, Wollo University, Dessie City, Ethiopia; 5 Department of Health Informatics, School of Public Health, College of Medicine and Health Sciences, Wollo University, Dessie City, Ethiopia

**Keywords:** Health care, Health occupations

## Abstract

Ensuring universal access to family planning services is a proven strategy to improve reproductive health as well as economic development. Assessing the trend and identifying the factors for the change in modern contraceptive utilization is crucial to design effective measures, but trend analysis was not conducted previously. Thus, this study aimed to assess the trend and determinants of modern contraceptive utilization change among married women in emerging regions of Ethiopia. This study used the 2000 Ethiopia Demographic and Health Survey and the 2019 Ethiopia Min Demographic and Health Survey datasets for analysis. A total of 2555 and 1916 married women in the 2000 and 2019 surveys were included in the analysis, respectively. The data were analyzed using Stata version 17.0. Logit-based decomposition analysis was executed to identify factors for modern contraceptive utilization change. Statistical significance was declared at a *P* value of less than 0.05. The trend of contraceptive utilization change increased from 6.26% in 2000 to 21.97% in 2019. About  − 65.87% and 165.87% of the change in contraceptive utilization was due to changes in composition and behavior, respectively. The change in composition was due to the change in the composition of women according to religion, educational status, region, and the number of living children. The change in behaviors of not educated women, rural women, Muslim women, and those who resided in the afar region was the source of change in modern contraceptive utilization. Modern contraceptive utilization has increased in the last two decades. The change in modern contraceptive utilization is due to changes in population composition and behavior. Interventions targeting uneducated and rural women are vital to increasing contraceptive utilization. Strategic interventions are also required for the Somali regions of Ethiopia.

## Introduction

Family planning has been associated with several benefits such as a reduction in maternal and infant mortality^1,2^. The utilization of hormonal contraceptives has been associated with various adverse effects, including changes in glucose levels^3^. Etonogestrel implants seem to be able to reduce pelvic pain and improve sexual function, and quality of life in patients with ovarian cysts suspected of endometriotic origin^4^. Globally, 44.2% of women of reproductive age (15–49 years) used a contraceptive method in 2019^5^. Globally, contraceptive use among women of reproductive potential who are married or in a union has risen from 55% in 1990 to 63% in 2020^6^. Contraceptive use among married/cohabiting women in sub-Saharan Africa increased from 13 to 33% over the same period and remains the lowest in the region^6^. In Ethiopia, modern contraceptive use rose from 6% in 2000 to 40% in 2019. However, this trend varies widely across regions, stagnating in the emerging regions of Ethiopia, where contraceptive use has increased by only 5% over 20 years. Added years^7,8^.

Family planning is one of the proven strategies to reduce maternal and child morbidity and mortality. It improves maternal health by reducing the number of pregnancies^9,10^ and avoiding unintended and closely spaced pregnancies^11^. Furthermore, if all unmet needs for modern contraception in developing regions were met, unintended pregnancies, unplanned births, and abortions would be reduced by about three-quarters. Comprehensive coverage of the unmet need for modern contraceptives would avert an estimated 76,000 maternal deaths per year^12^. Family planning also reduces child morbidity and mortality by avoiding too many and ill-timed pregnancies^13,14^.

The use of modern contraceptives is related to many factors. This includes; age^15,16^, marital status^16–18^, religion^16,19,20^, residence^21,22^, educational status^16,20–25^, wealth index^16,21,22,25^, media exposure^22^, discussion on family planning with a partner^18,19,26–28^, knowledge on modern contraceptive methods^15,23,29^, ever use of a modern contraceptive method^17,19^, number of alive children^20–22^, and the desired number of children^15,21,23^.

Investment in family planning is critical to achieving most of the Sustainable Development Goals (SDGs), mainly SDG 3 and SDG 5^30^. Because of this, several governmental and non-governmental organizations have made enormous efforts to increase access to family planning services. The Government of Ethiopia has set a target of increasing contraceptive prevalence to 55% by 2020^31^. However, only 41% of married women used birth control in 2019. However, expanding access to contraceptives remains a major challenge.

The emerging regions of Ethiopia are characterized by cultural, social, and economic diversity, which may influence the utilization of modern contraceptives. Therefore, understanding the determinants that drive the use of modern contraceptives in these regions is important to tailor interventions that target specific issues related to contraceptive utilization. Assessing the trends of modern contraceptive utilization and identifying contributing factors for the change is vital to design appropriate interventions to increase family planning service utilization. Most studies conducted on modern contraceptive utilization were only localized to certain settings and their sample size was too small^19,23,27,28^. Moreover, those studies which used Ethiopia Demographic and Health Survey (EDHS) data did not assess determinants of change in contraceptive utilization^32^. Thus, this study aimed to assess the trend and determinants of modern contraceptive utilization change among married women in the emerging region of Ethiopia. The study suggests that efforts should be made to increase access and education about modern contraceptive methods in regions where utilization is low. Additionally, addressing compositional factors such as educational status and religion may also contribute to increasing utilization. These findings could inform policy and program development aimed at improving reproductive health outcomes for married women in emerging regions of Ethiopia.

## Material and methods

### Study area and population

The study was conducted in emerging regions of Ethiopia, which are the Afar, Somali, Benshangul Gumz, and Gambela regions. Ethiopia is one of the Sub-Saharan African countries. This study used 2000 and 2019 EDHS data sets, which were collected by the Central Statistical Agency (CSA) in coordination with the Federal Minister of Health (FMOH) and the Ethiopia Public Health Institute (EPHI). The Ethiopia Population and Housing Census were used as a sampling frame and a stratified multistage sampling technique was used. A total of 2555 and 1916 married women in 2000 EDHS and 2019 MEDHS were included for analysis, respectively.

### Variable measurement

The dependent variable of modern contraceptive utilization was classified dichotomously as "Yes/No". Married women who used a modern contraceptive method during the time of the interview were categorized as "Yes" and who did not use a modern contraceptive method during the time of the interview were categorized as "No".

### Data quality control and analysis

First exploratory data analysis was done to check for outliers, missing, and consistency. All results were weighted for sampling probabilities using the weighting factor in the EDHS data. SVY STATA command was used to control the clustering effect of complex sampling. The data were analyzed by using Stata version 17.0. The two datasets were merged by using the Stata command “append”. Multivariate decomposition analysis was performed to see the change in modern contraceptive utilization and its contributing factors.

The logistic regression model output was used to decompose the observed difference in modern contraceptive utilization into two components. The first change was due to variation in population composition across the survey. The second change was due to a change in the behavior of the population. Thus, the change in outcome was due to either change in population composition or a change in the behavior of the population, or both. The observed differences in modern contraceptive utilization between the two surveys were decomposed into characteristics (population composition) and a coefficient (effect of characteristics).

### Ethical approval and consent to participate

Ethical approval was obtained from the Ethiopia Demographic and Health Survey (EDHS) dataset for research purposes. The EDHS is a nationally representative survey conducted by the Ethiopian Central Statistical Agency (CSA), with the support of various international partners, including ICF International, USAID, and UNFPA. The data were accessed from CSA by requesting it through the website www.measuredhs.com. Then, an authorization letter was received from CSA to download the EDHS dataset. The data were used only for this study and it was not passed to other researchers. All data were treated as confidential and no personal or household identifiers were used in the survey. The IRB-approved procedures for DHS public-use datasets do not in any way allow respondents, households, or sample communities to be identified. Detailed information on ethical issues was found within the EDHS report. All methods and materials were carried out under relevant guidelines and regulations.

## Result

### Characteristics of the respondents

The mean age of respondents was 30.21 years (± 0.17) and 29.18 years (± 0.18) in 2000 and 2019, respectively. The proportion of rural dwellers increased from 90.06% in 2000 to 82.36% in 2019. Concerning educational status, the proportion of women who were not educated decreased from 85.71% in 2000 to 60.02% in 2019. The majority of people reside in the Benshangul Gumz region and 27.82% in 2000 to 27.66% in 2019 (Table 1).

**Table 1:** Distribution characteristics of participants in 2000 EDHS and 2019 EMDHS.

Category	EDHS 2000, n = 2555% (95% CI)	EDHS, n = 1916% (95% CI)
Age		
15–24 years	27.4 (25.73, 29.20)	29.38 (27.38, 31.47)
25–34 years	40.08 (38.19, 41.99)	42.48 (40.29, 44.71)
35–49 years	32.48, (30.69, 34.33)	28.13 (26.16, 30.19)
Residence		
Urban	9.94 (8.84, 11.16)	17.64 (15.99, 19.41)
Rural	90.06 (88.84 91.16)	82.36 (80.58 84.03)
Religion		
Orthodox	19.06 (17.58, 20.63)	17.07 (15.45, 18.82)
Muslim	59.14 (57.22, 61.03)	64.20 (62.02, 66.31)
Protestant	15.12 (13.77,16.55)	16.65 (15.05, 18.40)
Other*	6.69 (5.79, 7.73)	2.09 (1.53, 2.83)
Educational status		
Not educated	85.71 (84.30, 87.02)	60.02 ( 57.81, 62.20)
Primary	11.08 ( 9.92, 12.35)	29.44 (27.44, 31.52)
Secondary and above	3.21 ( 2.59, 3.97)	10.54 ( 9.24, 12.00)
Region		
Afar	24.22 (22.64, 25.93)	25.16 ( 23.26, 27.15)
Somali	22.27 (20.70, 23.93)	23.28 (21.44, 25.22)
Benshangul Gumz	27.82 (26.12, 29.60)	27.66 (25.70, 29.71)
Gambela	25.68 (24.02, 27.41)	23.90 ( 22.05, 25.87)
Ever give birth		
Yes	88.38 (87.07, 89.56)	89.56 (88.81, 90.85)
No	11.62 (10.44, 12.93)	10.43 (9.14, 11.89)
Number of living children		
0	14.72 (13.39, 16.14)	11.84 (10.47, 13.37)
1–5	70.41 (68.61, 72.15)	67.33 (65.19, 69. 39)
≥ 6	14.87 (13.54, 16.31)	20.82 (19.06, 22.70)

*-Catholic and traditional religion follower.

### The trend of modern contraceptive utilization

The trend of contraceptive utilization among married women significantly increased from 6.26%, 95% CI (5.38, 7.27) in 2000 to 21.97% (20.17, 23.88) in 2019.

### The trend of modern contraceptive utilization showed different characteristics of respondents

Utilization of modern contraceptives was increased by 21.74% and 15.75% among 15–24 and 25–34 years, respectively. The trend of modern contraceptive utilization among rural dwellers increased by 17.21%. Modern contraceptive utilization among not educated women also increased from 3.61% in 2000 to 13.65% in 2019. Modern contraceptive utilization among women in less Benshangul Gumz region of Ethiopia increased by only 0.63 percentage points in the past two decades (Table 2).

**Table 2:** The trend of modern contraceptive utilization among married women by characteristics of respondents, 2000 EDHS and 2019 EMDHS.

Category	EDHS 2000, (n = 2555)	EMDHS 2019, (n = 1916)	Percentage difference (2019–2000)
Age			
15–24 years	7.13	28.87	21.74
25–34 years	7.71	23.46	15.75
35–49 years	3.73	16.70	12.97
Residence			
Urban	23.22	23.96	0.74
Rural	4.34	21.55	17.21
Religion			
Orthodox	14.58	52.29	37.71
Muslim	3.91	13.01	9.1
Protestant	7.25	25.71	18.46
Other*	1.17	20	18.83
Educational status			
Not educated	3.61	13.65	10.04
Primary	16.61	33.51	16.9
Secondary and above	41.46	37.13	− 4.33
Region			
Afar	5.65	10.99	5.34
Somali	2.28	2.91	0.63
Benshangul Gumz	7.45	36.60	29.15
Gambela	8.99	35.15	26.19
Ever give birth			
Yes	6.42	21.91	15.49
No	5.05	22.5	17.45
Number of living children			
0	5.85	21.59	15.74
1–5	6.89	25.27	18.38
≥ 6	3.68	11.53	7.85

*-Catholic and traditional religion follower.

### Decomposition analysis

The decomposition analysis model was performed and had taken an account the differences in the characteristics (compositional factors) and the differences due to the effect of characteristics (coefficient). About 65.87% of the overall modern contraceptive utilization change was due to differences in characteristics. Among the compositional factors, a significant contribution to change in modern contraceptive utilization among married women was due to religion, educational status, region, and the number of living children. The change in the composition of women who were not educated showed a significant contribution to increasing modern contraceptive utilization. The change in the composition of who were protestant and other religious follower women, Afar region resident women also showed a significant contribution to increasing modern contraceptive utilization. However, the change in the composition of Muslim women living in Somali regions contributed to a decrease in modern contraceptive utilization. Furthermore, a significant decrease in modern contraceptive utilization was due to the change in the composition of women who had one to five live children.

After controlling the effect of compositional factors, 165.87% of the change in modern contraceptive utilization was due to the difference in the effects of characteristics. A significant increase in modern contraceptive utilization was due to educational status, residence, and the number of living children. Change in behavior of uneducated and primary school, rural women, and women who had one to five alive children showed a significant increase in modern contraceptive utilization. (Table 3).

**Table 3:** Decomposition of change in modern contraceptive utilization among married women in Ethiopia, 2000 to 2019.

	Difference due to characteristics (E)			Difference due to coefficients (C)		
	Coefficient	Percent	*P* value	Coefficient	Percent	*P* value
Age						
15–24 years	1					
25–34 years	− 0.00013	− 0.26	0.838	0.00494	10.13	0.640
35–49 years	0.00070	1.43	0.494	0.00067	1.38	0.956
Place of residence						
Urban	1					
Rural	0.00266	5.45	0.284	0.04760	97.67	0.038
Religion						
Orthodox	1					
Muslim	− 0.01349	− 27.68	0.002	− 0.01311	− 26.91	0.313
Protestant	0.00353	7.25	0.000	− 0.01899	− 38.97	0.050
Other*	0.00314	6.45	0.007	− 0.00010	− 0.20	0.969
Education status						
Not educated	0.01167	23.95	0.000	0.11656	21.855	0.001
Primary	− 0.00189	− 3.88	0.411	0.00675	1.253	0.040
Secondary and above	1					
Region						
Benshangul Gumz	1					
Somali	− 0.04681	− 96.05	0.000	0.00399	8.19	0.716
Afar	0.00925	18.99	0.012	0.01689	34.66	0.050
Gambela	0.00031	0.64	0.390	0.00442	9.07	0.153
Ever give birth						
Yes	− 0.00002	− 0.05	0.290	− 0.11875	− 243.65	0.108
No	1					
Number of living children						
0	1					
1–5	− 0.01230	− 25.24	0.009	0.10929	224.24	0.041
≥ 6	0.01127	23.13	0.054	0.02422	49.70	0.095
Total	− 0.03210	− 65.87	0.000	0.08084	165.87	0.000

## Discussion

The trend of modern contraceptive utilization among married women of reproductive age significantly increased over the last two decades (2000–2019). This change was due to both differences in population composition (65.87%) and changes in the behavior of the population (165.87%).

Modern contraceptive utilization among married women of reproductive age increased from 6.26%, 95% CI (5.38, 7.27) in 2000 to 21.97% (20.17, 23.88) in 2019, or 15.71 percentage points. This increase is lower than the trend of modern contraceptive utilization in Sub-Saharan Africa by 20 percentage points^6^ but higher than the trend of modern contraceptive utilization Worldwide by 2.1 percentage points^5^. However, the trend of modern contraceptive utilization in emerging regions of Ethiopia greatly varies across the region ranging from a 0.63 percentage point increase in the Somali region to a 29.15% increase in the more Benshangul Gumz region of Ethiopia.

The change in modern contraceptive utilization was due to compositional factors and the effect of characteristics. Among the compositional factors, a significant contribution to change in modern contraceptive utilization among married women was due to religion, educational status, region, and the number of living children. Religion was an important compositional factor affecting change in modern contraceptive utilization. The change in the proportion of Muslim women had a negative significant effect on modern contraceptive utilization. The possible reason might be due to the individual's religious view held against contraceptive use.

The decrease in the proportion of women who were not educated and in primary school showed a significant contribution to increasing modern contraceptive utilization. This finding is in line with studies conducted in Ethiopia^23^, Ghana^18^, Liberia^20^, Senegal^21^, East Africa^16^, Sub-Saharan Africa^22^, and Africa^25^. Ensuring inclusive and equitable education is one of the key sustainable development goals and is emphasized by global, regional, and local organizations^33^. Due to this, the proportion of educated mothers has increased^34^. Educated mothers are exposed to contraceptive-related information and education. Furthermore, they used contraceptive methods due to fear of school dropout and the burden of raising children. Additionally, education has been shown to increase women's autonomy and decision-making power within their households, which may make it easier for them to negotiate contraceptive use with their partners. Therefore, as the proportion of educated women increases, it is likely that more women will choose to use modern contraceptives, which may help to reduce the incidence of unintended pregnancies and associated adverse effects, such as changes in glucose levels.

The increase in the proportion of women in Somali regions significantly decreased the change in modern contraceptive utilization. This might be due to women in the Somali regions strongly holding traditional beliefs which hinder modern contraceptives. Moreover, the proportion of educated and employed women is lower in Somali regions compared to other regions of Ethiopia.

The decrease in the proportion of women who had one to five children significantly decreased the change in modern contraceptive utilization. This might be due to women who had one to five children having better information and behavior change regarding contraceptive use. Women who have already had children may have gained more experience and knowledge about contraceptive use, leading to better information and behavior change regarding contraception. These women may also be more aware of the potential risks and benefits of different contraceptive methods and may be more likely to make informed decisions about their use.

About 165.87% of the change in modern contraceptive utilization was due to the difference in the effects of characteristics. A change in the behavior of rural women showed a significant increase in modern contraceptive utilization. The behavioral change in rural women might be due to the effect Health Extension Program that has been implemented since 2003 to achieve universal coverage of primary health care among the rural population in Ethiopia^35^. Family planning is one of the key health packages of the health extension program. The limitation of cultivated land and natural resources and the burden of raising children may also push rural women to use contraceptive methods to limit the number of children^36^.

A change in the behavior of uneducated and primary school women had a significant increase in modern contraceptive utilization. This might be also due to the effort made by community health workers who are working at the household level to improve health service utilization including family planning^35^. Married women who reside in the Afar region showed a significant increase in modern contraceptive utilization. Afar regions have better infrastructure, education, and healthcare access compared to the others region.

This study utilized large datasets and considered sampling weighing during analysis. However, since the two surveys were not conducted with the same respondents, it is not a real-time series analysis. Additionally, this analysis considers a few variables recorded in both surveys, which are not the only factors that affect modern contraceptive utilization.

## Conclusions

Modern contraceptive utilization among married women in emerging regions of Ethiopia increased in the past two decades. The trend significantly varies across regions of Ethiopia. The change was due to both the change in population composition and behavior. Women's religion, educational status, residence, region, and the number of living children were compositional and behavioral factors affecting change in modern contraceptive utilization. Interventions targeting uneducated and rural women are important to increase contraceptive utilization. Strategic interventions are also required for the Somali regions of Ethiopia.

## Data Availability

The data will also be made available from corresponding author on reasonable request.

## Abbreviations

CSA: Central statistical agency
EDHS: Ethiopia demographic, and health survey
EMDHS: Ethiopia min demographic, and health survey
EPHI: Ethiopia public health institute
FMOH: Federal ministry of health
SDG: Sustainable development goals
